# Teledermatology and daylight photodynamic therapy: Managing actinic keratoses in the elderly in nursing homes in Spain

**DOI:** 10.1016/j.jdin.2025.06.008

**Published:** 2025-08-12

**Authors:** Paola Pasquali, Maria Luz Negrín, Ana María Rodríguez, Natalia Rovira González, Silvana Ballester Bellmunt, Mioara Madalina Diaconu

**Affiliations:** a Department of Dermatology, Pius Hospital of Valls, Valls, Spain; b Department of Medicine and Medical Specializations, University of Alcalá, Madrid, Spain; c Centro Médico Pasquali y Asociados, Cambrils, Spain; d Residencia de Gent Gran Alt Camp, Tarragona, Spain; e Residencia Santa Teresa, Valls, Spain

**Keywords:** actinic damage, daylight PDT, nursing home, PDT, photodynamic therapy, skin cancerization, teledermatology

*To the Editor:* In Spain, projections for 2040 estimate that nearly 27% of the population will be >65 years old. The longer life expectancy, together with continuous sun exposure over the years, has been related to an increased incidence of actinic keratosis (AK) and keratinocytic tumors.^1^ In this context, teledermatology plays a key role, facilitating early diagnosis and, consequently, early treatment.^2^^,^^3^ The synergy between telematic assistance and remote management of patients treated with therapeutic options such as daylight photodynamic therapy could be an excellent possibility for patients with reduced mobility, especially those living in nursing homes.^4^^,^^5^

We conducted an observational prospective study in 2 nursing homes in Spain, focused on elderly patients (>65 years old) with AK grade I or II. Clinical and dermatoscopic images were taken and sent from the nursing home to the physician using a teledermatology/teledermoscopy medical device by DeepX Health. These images were checked, and the diagnosis of AK was confirmed by a dermatologist. Afterward, a single treatment session with the following daylight photodynamic therapy protocol was prescribed. After curettage and chemical sunscreen application, a nurse applied methyl aminolevulinate 160 mg/g cream to the area with AK. Following a 30-minute incubation period, patients were exposed to daylight for 2 hours. After exposure, the product was removed with water; sun protection factor 50+ sunscreen was applied, and patients were advised to use moisturizing creams for 1 week following treatment and to avoid sun exposure during the following 48 hours. Treatment effectiveness was assessed at the 3-month follow-up visit. Complete response was defined as complete disappearance of lesions, while partial/no response included partial, minimal, or no improvement of lesions.

A total of 26 patients, 79% female, participated in the study. All the AK lesions treated were located on the facial area. Clinical evaluations indicated that 57.7% of patients achieved complete response, while 42.3% showed partial or no improvement (Fig 1, *A*). On the other hand, dermatoscopic assessments identified complete response in 34.6% of patients, with 65.4% showing partial/no response (Fig 1, *A*). When examining the combination of clinical and dermatoscopic results (Fig 1, *B*), 35% exhibited complete response in both assessments, while 42% showed partial/no response across both metrics. Additionally, 23% achieved complete clinical response alongside partial/no dermatoscopic response. Remarkably, no cases with partial/no clinical response were found to correspond with complete dermatoscopic response. Moreover, an overall clinical enhancement in skin condition was reported, highlighted by a notable reduction in signs of photoaging and actinic damage, contributing to a visibly healthier appearance that was appreciated by both the patients and the clinicians involved in this study. There were no complications reported, and no significant safety issues were observed during the study.

**Fig 1:**
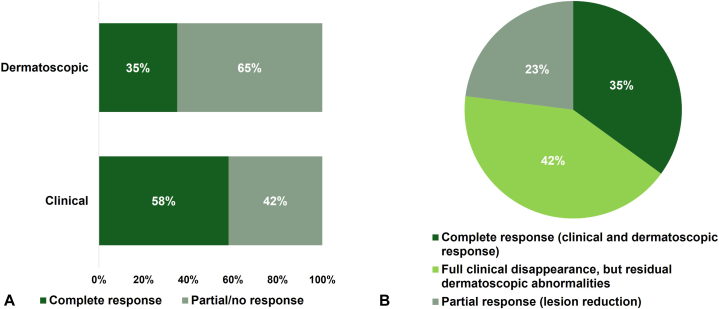
Actinic keratosis. **A,** Dermatoscopic and clinical response to treatment. **B,** Complete response is defined as complete disappearance of lesions.

In conclusion, the combination of teledermatology and daylight photodynamic therapy offers a practical and effective solution for managing AK in elderly populations, particularly in nursing homes. This approach not only enhances patient care by providing timely and less invasive treatment options but also aligns with health care strategies aimed at reducing costs and improving accessibility.

## Conflicts of Interest

Dr Pasquali is on the advisory board of DeepX Diagnostics Inc. Drs Negrín and Rodríguez and authors González, Bellmunt, and Diaconu have no conflicts of interest to declare.
